# Development and Validation of a High-Performance Liquid Chromatographic Method for the Determination of Cinitapride in Human Plasma

**DOI:** 10.1155/2018/8280762

**Published:** 2018-08-28

**Authors:** Boovizhikannan Thangabalan, Getu Kahsay, Tadele Eticha

**Affiliations:** School of Pharmacy, College of Health Sciences, Mekelle University, Mekelle, Ethiopia

## Abstract

A precise and reliable reversed-phase high-performance liquid chromatographic method with ultraviolet detection was developed and validated to determine cinitapride in human plasma. After liquid-liquid extraction, chromatographic separation was achieved on a Nucleosil C18 (25 cm × 4.6 mm, 5 *µ*m) column with an isocratic elution consisting of 10 mM ammonium acetate (pH 5.2), methanol, and acetonitrile, 40 : 50 : 10, v/v/v. The developed method was validated as per US FDA guidelines for its linearity, selectivity, sensitivity, precision, accuracy, and stability. Satisfactory findings were obtained from the validation studies. The linearity range of the method was 1 to 35 ng/mL while the extraction recovery of cinitapride in human plasma was more than 86%. The percent coefficient of variation of both intraday and interday precision was ≤7.1%.

## 1. Introduction

**Figure 1:**
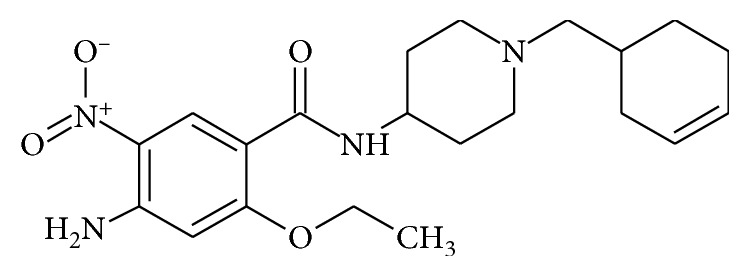
Chemical structure of cinitapride.

Cinitapride (Figure 1), chemically 4-amino-N-[3-(cyclohexan-1-yl-methyl)-4-piperidinyl]-2-ethoxy-5-nitrobenzamide, is a substituted benzamide gastroenteric prokinetic agent acting via complex, but synergistic effects on serotonergic 5-HT2 (inhibition) and 5-HT4 (stimulation) receptor and dopaminergic D2 (inhibition) receptors in the neuronal synapses of the myenteric plexi [1–3].

Several analytical methods have been reported for the determination of cinitapride in biological samples, pure and pharmaceutical dosage forms using various methods. A survey of literature revealed UV spectrophotometric methods [4, 5], extractive spectrophotometric estimation [6], colorimetric estimation [7] in formulation, polarographic method [8], liquid chromatography-tandem mass spectrometry (LC-MS/MS) methods for its determination in plasma [9], and reversed-phase high-performance liquid chromatography (RP-HPLC) method [10] in bulk drug.

The present study reports analysis of cinitapride in human plasma using the Nucleosil C18 column following a liquid-liquid extraction of the sample. The purpose of this study was to develop a rapid, economical, precise, and accurate RP-HPLC method for the determination of cinitapride in human plasma.

## 2. Experimental

### 2.1. Materials

Cinitapride was provided by Zydus research laboratories limited (Ahmedabad, India). All analytical grade ammonium acetate, triethyl amine, HPLC grade methanol, and acetonitrile were purchased from Merck (Mumbai, India) while high purity water was prepared using a Milli-Q water purification system (Mumbai, India). Isolated human plasma obtained from Lion's blood bank (Guntur, India) and stored in a freezer.

### 2.2. Instrumentation and Chromatographic Conditions

HPLC experiments were performed on a Shimadzu HPLC system (Shimadzu, Japan) equipped with Nucleosil C18 (25 cm × 4.6 mm, 5 *µ*m) column, LC2010 series pump, manual Rheodyne injector (with 20 *µ*L loop size), SPD-20A UV-visible detector, and Spinchrom software was used. The mobile phase consisted of 10 mM ammonium acetate buffer (adjusted to pH 5.2 with triethyl amine), methanol, and acetonitrile in the ratio of 40 : 50 : 10 v/v/v, that was set at a flow rate of 1 mL/min.

### 2.3. Preparation of Standard Solution

A stock solution was prepared by dissolving accurately weighed 100 mg of cinitapride in 100 mL of HPLC-grade methanol to yield a final concentration of 1 mg/mL of the drug. The stock solution (1000 *µ*g/mL of cinitapride) was diluted suitably and spiked with human blank plasma to get 1 to 35 ng/mL of drug.

### 2.4. Extraction of Drug from Plasma

Five hundred microliters of each standard solution (drug spiked human plasma) was pipetted into a series of polypropylene tubes and vortexed briefly. Three milliliters of *tert*-butyl methyl ether was added to each tube and caped. All calibration samples were vortexed for approximately for 10 minutes and centrifuged at 4000 rpm for approximately 5 minutes at 10°C. The standard supernatant layer was decanted into each clean polypropylene tube and evaporated to dryness at 40°C under a stream of nitrogen. Then, the dried extract was reconstituted in 500 *μ*L of mobile phase and a 20 *μ*L aliquot was injected into the chromatographic system using Hamilton syringe.

### 2.5. Validation

The developed method was validated as per US FDA guidelines [11] for its linearity, selectivity, sensitivity, precision, accuracy, and stability during various stress conditions including bench-top stability, freeze-thaw stability, stability of stock solutions, and dilution integrity.

#### 2.5.1. Selectivity

The selectivity of the proposed method was examined by analyzing the human blank plasma for endogenous interference. The absence of interfering peaks at the same retention time of cinitapride was considered as evidence for selectivity of the method.

#### 2.5.2. Linearity

Linearity was studied by spiking blank plasma samples with appropriate volume of stock solutions of cinitapride yielding 1 to 35 ng/mL. Calibration data were generated by injecting extracted solutions of the drug into the HPLC system.

#### 2.5.3. Precision and Accuracy

For precision and accuracy studies, samples were prepared at three concentration levels: low (LQC), medium (MQC), and high (HQC) quality controls, corresponding to 3, 15, and 35 ng/mL of cinitapride, respectively, with six replicates each. The intraday and interday precision were determined by analyzing the prepared samples on the same and three different days, respectively. Precision was characterized by the percent coefficient of variation (%CV) whereas accuracy was expressed as a percent recovery of the drug.

#### 2.5.4. Recovery

Recovery of cinitapride was evaluated by comparing the detector response of cinitapride in three quality control samples (LQC, MQC, and HQC) with the response of the same concentration in methanolic solutions.

#### 2.5.5. Sensitivity

The limit of detection (LOD) and quantification (LOQ) were calculated from a calibration curve constructed using solutions containing a cinitapride in the range of detection limit.

#### 2.5.6. Stability: Bench-Top Stability

Six aliquots each of the three quality control samples were kept at room temperature (25 ± 5°C) after spiking into human plasma. After completion of 6 hrs, the samples were extracted and analyzed against the concentration of a freshly prepared one. Percent changes (bias) for cinitapride concentration for spiked samples over stability testing period of 6 hrs at room temperature was determined and compared to nominal values.

*(1) Stock Solution Stability*. The short-term stock solutions stability of analyte was evaluated at room temperature (25 ± 5°C) for at least 6 hrs. Long-term stability of analyte was evaluated at refrigerated temperature (2–8°C) for 35 days by comparing instrument response of the stability samples to that of comparison samples. Percent change (bias) in cinitapride concentration over the stability testing period of 6 hrs at 25°C was determined.

*(2) Freeze and Thaw Stability*. The freeze and thaw stability of analyte was determined after at least three freeze and thaw cycles. At least six aliquots at each of three quality control samples were stored at −20 ± 5°C and subjected to three freeze thaw cycles at an interval of 8–16 hrs. After the completion of third cycle, the samples were analyzed and the stability of samples were compared against freshly prepared calibration curve samples. Percent change (bias) in cinitapride concentration over the stability-testing period after three freeze-thaw cycles was determined.

*(3) Dilution Integrity*. Sample having final concentration about two times of higher calibration curve standard was prepared in plasma. Then, the samples were diluted five times and ten times with analyte-free control human plasma to meet their actual concentrations in the calibration curve range. The samples were extracted, and results were compared with nominal concentration.

## 3. Results and Discussion

### 3.1. Method Development and Optimization

During HPLC method development, different options were examined to optimize detection parameters, chromatography conditions, and sample extraction. The UV detector was used for the estimation of cinitapride at 260 nm to maximize the signal of the drug and minimize the signal of plasma interferents.

**Figure 2:**
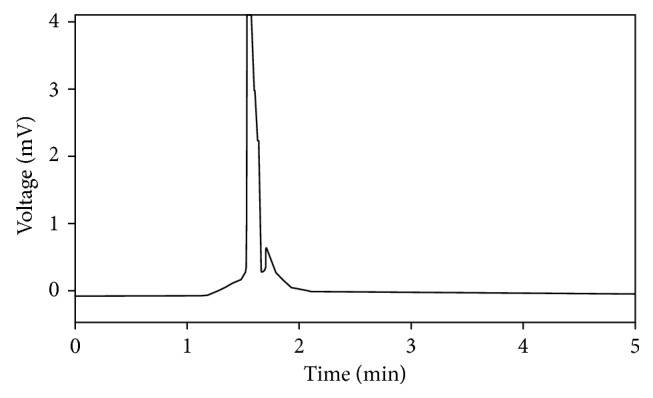
A typical chromatogram of blank plasma.

**Figure 3:**
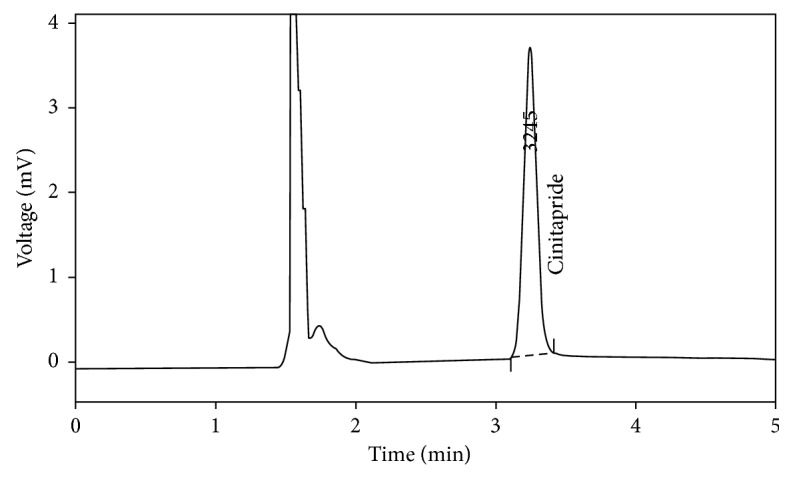
A typical chromatogram of cinitapride in human plasma.

The compositions of mobile phase were optimized through several trials to achieve good resolution and symmetric peak shape for the drug. Optimization of HPLC conditions performed on chromatographic parameters including retention time, column efficiency (HETP) of the various compositions, and velocity of mobile phase. Efficiency values (*N*) showed the results of ≥5600, and this suggested that the sharp peaks produced is enough. Cinitapride was eluted at 3.25 minutes. The typical chromatograms for the blank plasma and sample are given in Figures 1 and 2, respectively. A typical chromatogram of cinitapride in human plasma is shown in Figure 3. The system suitability parameters are given in Table 1.

### 3.2. Validation

#### 3.2.1. Selectivity

Selectivity was evaluated by extracting different blank plasma samples. The absence of interfering peaks at the retention time of cinitapride was considered as evidence for selectivity of the method. A chromatogram obtained from the blank plasma preparations indicates that there is no any interference of plasma components at the retention time of the drug. Chromatograms of both blank plasma and cinitapride in plasma are given in Figures 1 and 2, respectively.

#### 3.2.2. Linearity

The peak areas of calibration standards were proportional to the amount of cinitapride over the concentration range of 1 to 35 ng/mL. The linearity of a calibration curve was proved by high correlation coefficient (*r*^2^ = 0.999) and the low value of the *y*-intercept of the regression equation.

#### 3.2.3. Recovery

Six replicates of three quality control samples were prepared for recovery determination, and the peak areas obtained were compared to the peak areas obtained from extracted samples of the same concentration levels. The percent recovery ranged from 86.6% to 99.93% (Table 2). The recovery obtained for cinitapride was within the range of 86.6% to 91.93% recommended by US FDA.

#### 3.2.4. Precision and Accuracy

The precision of the developed method was evaluated as intraday and interday precision. They were examined by analyzing six replicates of three quality control samples on the same day and three consecutive days. The percent coefficient of variation of both intraday and interday precision was ≤7.1% (Table 3). Furthermore, the accuracy was evaluated from the same quality control samples, and the percent recovery ranged from 86.04% to 93.88% (Table 3).

#### 3.2.5. Sensitivity

The LOD and LOQ determined for cinitapride were 0.1192 and 0.3612 ng/mL, respectively. The values obtained were satisfactory. All the results for validation parameters are summarized in Table 4.

#### 3.2.6. Stability

Bench-top, short-term stability, long-term stability, and freeze-thaw cycle for cinitapride were investigated at different quality control samples. The results revealed that cinitapride was stable in plasma for at least 6 hrs at room temperature and 35 days at 2–8°C. It was approved that repeated freezing and thawing (three cycles) of plasma samples spiked with cinitapride at all levels did not influence their stability. In addition, dilution integrity confirmed that dilution of the samples with analyte-free control human plasma did not affect the stability. The findings obtained from all these stability studies are shown in Table 5.

## 4. Conclusion

Optimization of RP-HPLC conditions and extraction of cinitapride from human blood plasma by liquid-liquid extraction have been performed and analyzed by the HPLC-UV method. The developed method was validated for its selectivity, linearity, precision, accuracy, and stability. The method can be used to analyze cinitapride in human blood plasma, so that the results obtained can be directly used to determine the bioavailability and its bioequivalence.

## Figures and Tables

**Table 1:** System suitability data.

Drug	USP plate count	Tailing factor (*T*)	Retention time (min) (*n*=6)		Peak area (*n*=6)	
Cinitapride 15 ng/mL	5602	1.05	Mean ± SD	%RSD	Mean ± SD	%RSD
			3.25 ± 0.01	0.28	493 ± 13	2.6

**Table 2:** Recovery study.

S. no.	LQC response		MQC response		HQC response	
	Extracted	Unextracted	Extracted	Unextracted	Extracted	Unextracted
1	110	116	459	543	1086	1170
2	94	119	496	549	1069	1176
3	116	120	483	551	1091	1182
4	89	114	492	542	1035	1172
5	92	109	469	549	1092	1169
6	100	116	491	551	1110	1183
Mean	100.16	115.66	481.67	547.50	1080.50	1175.33
SD	10.74	3.93	14.66	3.99	25.87	6.05
% CV	10.72	3.40	3.04	0.73	2.39	0.51
% recovery	86.60		87.98		91.93	

LQC, 3 ng/mL; MQC, 15 ng/mL; HQC, 35 ng/mL of cinitapride.

**Table 3:** Precision and accuracy of cinitapride.

Concentration (ng/mL)	Accuracy (%nominal) (*n*=6)	Precision (%CV)	
		Interday (*n*=18)	Intraday (*n*=6)
3	86.04	5.57	7.10
15	90.40	4.68	3.04
35	93.88	2.03	4.35

**Table 4:** Validation parameters.

Parameters	Results
Selectivity	Pass
System suitability	Pass
Linearity (ng/mL)	1–35
Range (ng/mL)	0.1–35
LOD (ng/mL)	0.1192
LOQ (ng/mL)	0.3612
Accuracy and precision	Pass
Short-term stock stability	Pass
Long-term stock stability	Pass
Long-term plasma stability	Pass
Bench-top stability	Pass

**Table 5:** Stability studies.

Stability	Spiked final concentration (g/mL)	Mean ± SD	%CV	%nominal	%RE
BT	3	2.79 ± 0.10	3.47	93.05	−6.95
	15	14.52 ± 0.22	1.51	96.79	−3.20
	35	33.83 ± 0.49	1.45	96.65	−3.35
ST	35	32.82 ± 0.68	3.61	93.78	−6.22
LT	35	33.22	2.52	94.90	−5.10
FT	3	2.78 ± 0.08	3.09	2.78	−7.22
	10	9.59 ± 0.25	2.63	95.90	−4.10
	25	24.46 ± 0.18	0.73	97.84	−2.16
DI	14	13.55 ± 0.28	2.04	96.80	−3.20
	7	6.82 ± 0.11	1.65	97.38	−2.62

BT: bench-top stability, 6 hrs; ST: short-term stability, 6 hrs; LT: long-term stability, 35 days; FT: freeze-thaw stability, 3 cycles; DI: dilution integrity.

## Data Availability

The data used to support the findings of this study are available from the corresponding author upon request.
